# Supplementary material for: Structural Mechanisms of DNAJC13 Dimeric Assembly and InsP6 binding in Recycling Endosome Regulation
Source: bioRxiv. 2026 Jul 29:2026.07.28.740790. Preprint. [Version 1] doi: 10.64898/2026.07.28.740790 (PMC13618431; doi:10.64898/2026.07.28.740790)
Supplement: Supplementary file 1 [file NIHPP2026.07.28.740790V1-supplement-1.pdf]

bioRxiv preprint doi: https://doi.org/10.64898/2026.07.28.740790; this version posted July 29, 2026. The copyright holder for this preprint (which was not certified by peer review) is the author/funder, who has granted bioRxiv a license to display the preprint in perpetuity. It is made available under a CC-BY 4.0 International license.

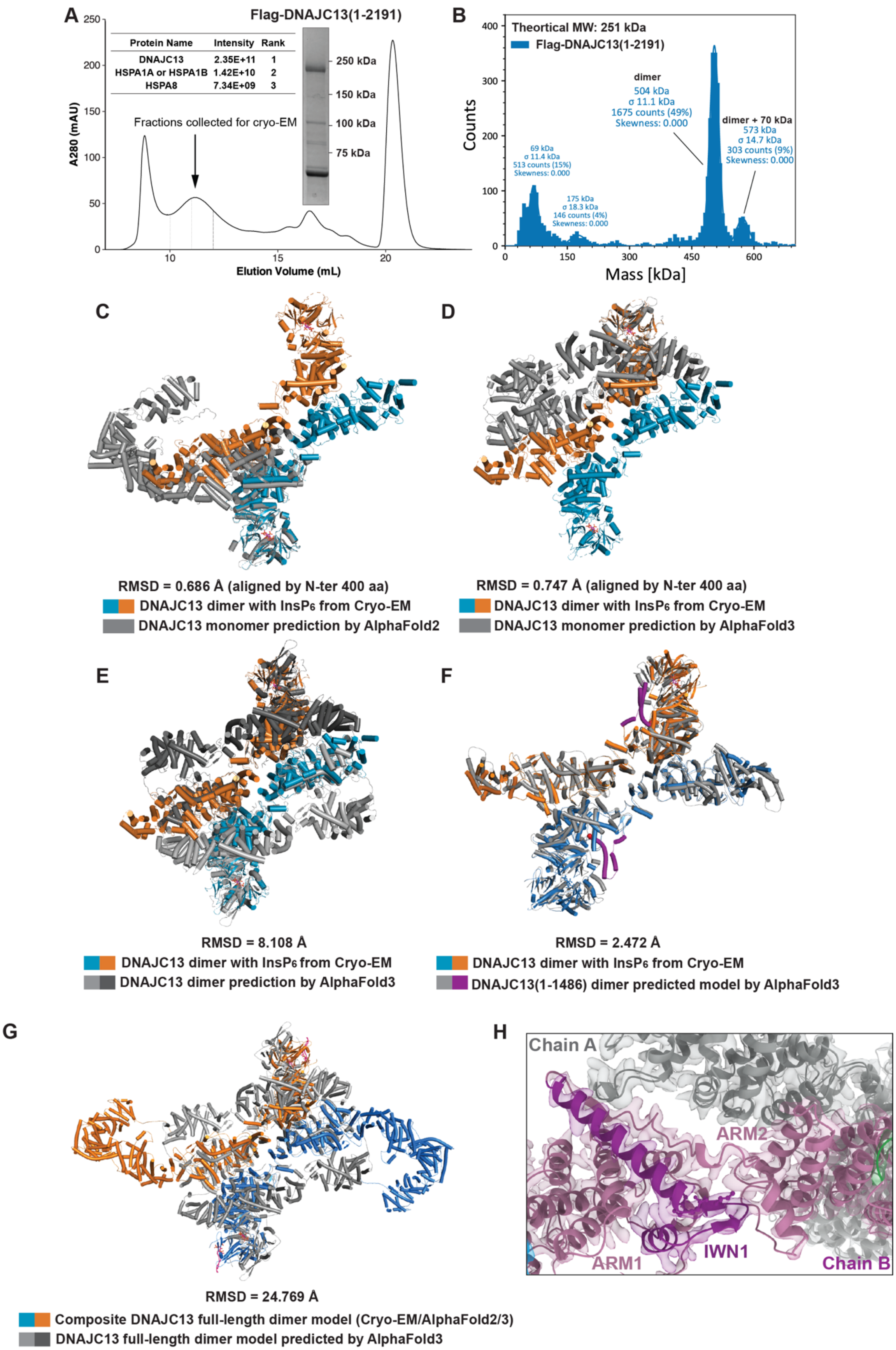

bioRxiv preprint doi: https://doi.org/10.64898/2026.07.28.740790; this version posted July 29, 2026. The copyright holder for this preprint (which was not certified by peer review) is the author/funder, who has granted bioRxiv a license to display the preprint in perpetuity. It is made available under a CC-BY 4.0 International license.

Figure S1. Structural Alignment and Comparative Analysis of the DNAJC13(1-1486) Cryo-EM Structure and AlphaFold Predictions, Related to Figure 1.
(A) Size-exclusion chromatography profile of Flag-DNAJC13(1-2191) purified from Expi293F cells. UV absorbance at 280 nm is plotted against elution volume. Inset, Coomassie-stained SDS-PAGE of the peak fraction. Size markers indicate mass in kDa. Inset: Mass-spectrometry analysis of purified DNAJC13(1-2191) from Expi293F cells. Proteins identified listed with their label-free intensities and abundance rank. DNAJC13 is the most abundant species, with HSPA1A or HSPA1B and HSPA8 detected as the most abundant co-purifying chaperones.
(B) Mass photometry of Flag-DNAJC13(1-2191) purified from Expi293F cells. The theoretical molecular weight of the monomer is 251 kDa. The major species corresponds to a dimer (504 kDa), with a minor population of dimer plus a 70-kDa complex, indicating that DNAJC13 forms a dimer in solution.
(C-F) Structural overlays of the experimentally determined DNAJC13(1-1486) homodimer cryo-EM model (colored in blue and orange) with corresponding predictive structural models (colored in gray): (Panel C) AlphaFold2 predicted monomer model, (Panel D) AlphaFold3 predicted monomer model, (Panel E) AlphaFold3 predicted full-length dimer model, and (Panel F) AlphaFold3 predicted DNAJC13(1-1486) dimer model.
(G) Structural overlays of the composite DNAJC13 homodimer model by merging Cryo-EM and AlphaFold2/3 (colored in blue and orange) with AlphaFold3 predicted DNAJC13 full-length dimer model.
(H) Packing of the IWN1 motif, a fold apparently unique to DNAJC13, with the subsequent ARM2 domain. Structural elements in Chain B are shown in magenta while chain A is shown in grey.

bioRxiv preprint doi: https://doi.org/10.64898/2026.07.28.740790; this version posted July 29, 2026. The copyright holder for this preprint (which was not certified by peer review) is the author/funder, who has granted bioRxiv a license to display the preprint in perpetuity. It is made available under a CC-BY 4.0 International license.

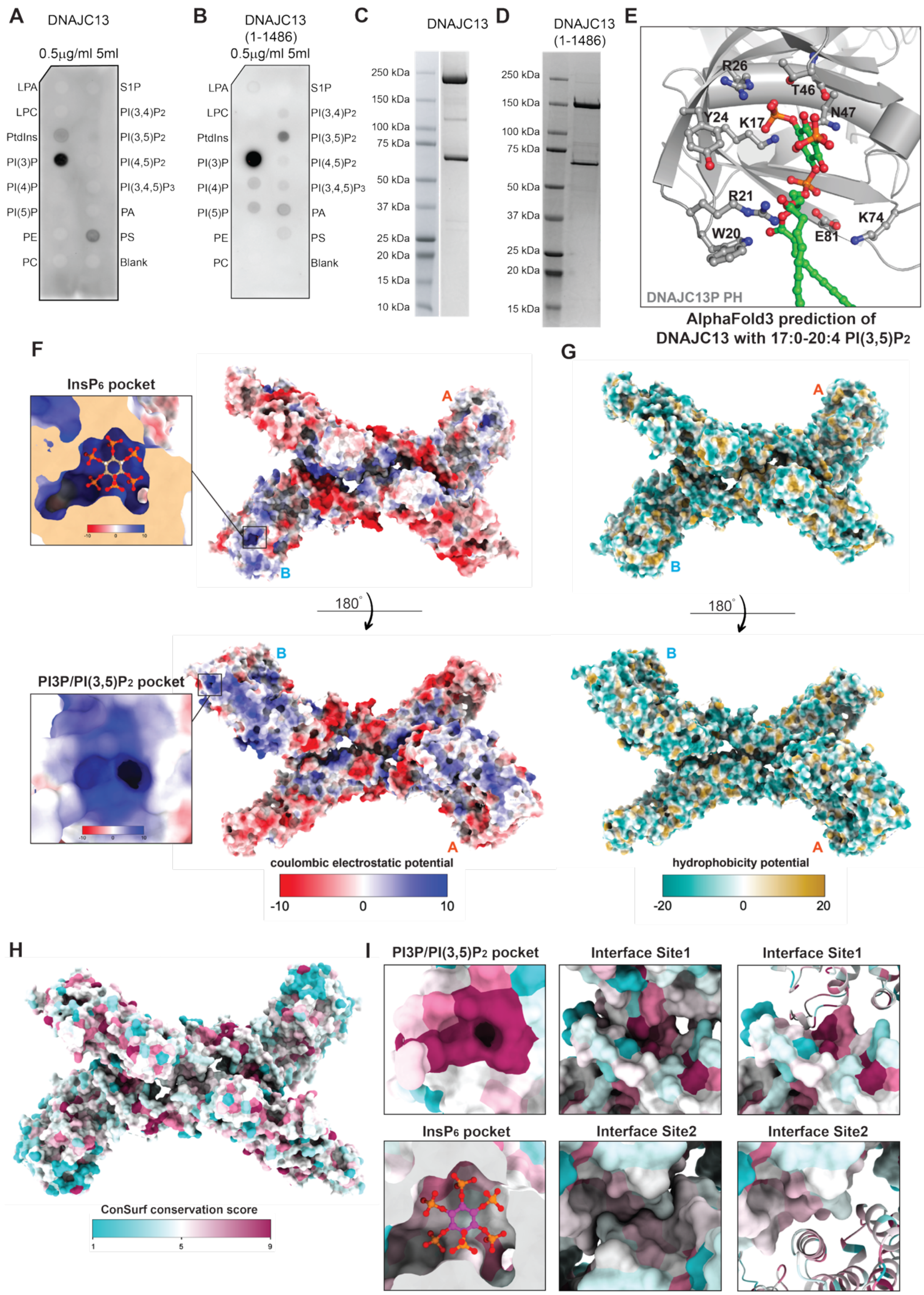

Figure S2. Surface properties, conservation, and phosphoinositide binding of DNAJC13, related to Figure 2.

bioRxiv preprint doi: https://doi.org/10.64898/2026.07.28.740790; this version posted July 29, 2026. The copyright holder for this preprint (which was not certified by peer review) is the author/funder, who has granted bioRxiv a license to display the preprint in perpetuity. It is made available under a CC-BY 4.0 International license.

(A, B) Lipid-protein overlay assays (PIP strips) using full-length DNAJC13 (Panel E) or DNAJC13(1-1486) (Panel F). Proteins (0.5 μg/ml) were incubated with membrane-spotted lipids as indicated, and bound protein was detected by immunoblotting, revealing preferential binding to PI3P.
(C, D) Coomassie-stained SDS-PAGE gels showing the purity of full-length DNAJC13 (Panel C) and DNAJC13(1-1486) (Panel D) used in the lipid-binding assays.
(E) AlphaFold3 predicted model of the DNACJC13 with 17:0-20:4 PI(3,5)P_2, illustrating the specificity of the PI3P-binding pocket. Key basic and polar residues forming the predicted binding are shown as sticks around the PI(3,5)P_2 headgroup.
(F) Coulombic electrostatic potential of the DNAJC13(1-1486) dimer cryo-EM model shown in two orientations related by 180°. Insets highlight the highly basic $InsP_6$ pocket formed by PHL2-PHL3 and the basic pocket on PHL1 that binds PI3P.
(G) Hydrophobicity surface of the same model and orientations as in (Panel A), colored from hydrophilic (cyan) to hydrophobic (yellow).
(H) Surface representation colored by ConSurf conservation scores, from variable (cyan) to highly conserved (magenta).
(I) Close-up views of conserved surface areas. Top left, conserved cavity corresponding to the PI3P pocket on PHL1. Bottom left, moderately conserved $InsP_6$ pocket between PHL2 and PHL3. Top middle/right, highly conserved patches at dimer interface Site 1 between the two protomers (surface and ribbon views). Bottom middle/right, intermediately conserved patches at dimer interface Site 2 (surface and ribbon views).

bioRxiv preprint doi: https://doi.org/10.64898/2026.07.28.740790; this version posted July 29, 2026. The copyright holder for this preprint (which was not certified by peer review) is the author/funder, who has granted bioRxiv a license to display the preprint in perpetuity. It is made available under a CC-BY 4.0 International license.

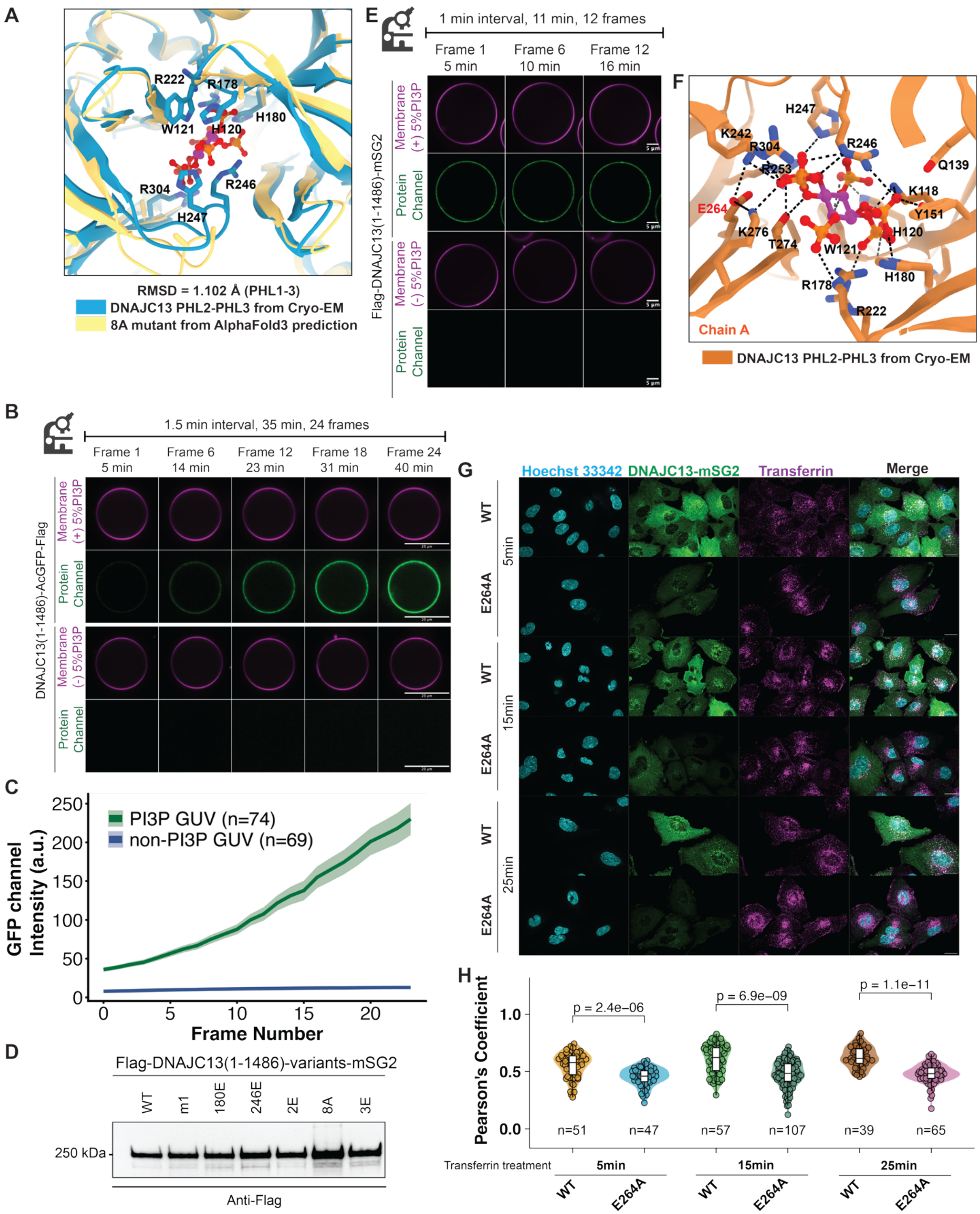

Figure S3. InsP6 binding is required for efficient recruitment of DNAJC13 to PI3P-containing membranes, related to Figure 3.

(A) Structural comparison of the PHL2-PHL3 $InsP_6$-binding pocket from the cryo-EM model (blue) and the 8A mutant predicted by AlphaFold3 (gold). Residues mutated in the 8A variant are shown as sticks at the corresponding positions in WT and 8A; the overall RMSD for PHL1-3 is indicated.

bioRxiv preprint doi: https://doi.org/10.64898/2026.07.28.740790; this version posted July 29, 2026. The copyright holder for this preprint (which was not certified by peer review) is the author/funder, who has granted bioRxiv a license to display the preprint in perpetuity. It is made available under a CC-BY 4.0 International license.

(B) Time-lapse confocal imaging of DNAJC13(1-1486)-AcGFP-Flag incubated with GUVs containing 5% PI3P or lacking PI3P. DNAJC13 accumulates robustly and progressively on PI3P-containing membranes but not on PI3P-deficient GUVs. Magenta, fluorescent lipid membrane dye; green, DNAJC13(1-1486)-AcGFP-Flag. Representative frames from a 35-min imaging series (1.5-min interval, 24 frames total) are shown. Scale bars, 20 μm.
(C) Quantification of protein binding to PI3P-containing (dark green) versus non-PI3P (light green) GUVs over time. GFP channel intensity at the membrane is plotted as mean ± SEM for the indicated number of GUVs (n).
(D) Immunoblot of input Flag-DNAJC13(1-1486)-mSG2 variant proteins used in GUV binding assays, probed with anti-FLAG antibody, confirming comparable protein levels for all constructs. Note: the blot contains variant proteins that were not employed in GUV binding assays.
(E) Time-lapse images of PI3P-containing GUVs incubated with DNAJC13(1-1486)-mSG2. Protein (green) rapidly and stably decorates the PI3P-positive membrane but not PI3P-deficient membrane (magenta). Images were acquired every 1 min for 11 min.
(F) Close-up view of the $InsP_6$-binding pocket in chain A of the cryo-EM structure, highlighting residues that coordinate $InsP_6$. E264 forms hydrogen bonds with K276 and K242 in PHL3, positioning these lysines to engage $InsP_6$ phosphates.
(G) Confocal images of SUM159 cells expressing DNAJC13-mSG2 WT or the E264A mutant (green, see **Data S3B** for immunoblots) after 5, 15, or 25 min of fluorescent Transferrin uptake (magenta). Nuclei are labeled with Hoechst 33342 (cyan). Compared with WT, E264A shows reduced punctate localization and diminished overlap with Transferrin-positive endosomes. Scale bars, 20 μm.
(H) Pearson's correlation analysis of DNAJC13-Transferrin colocalization for WT and E264A at the indicated time points. Violin plots show the distribution of Pearson's coefficients with median and interquartile ranges; n indicates the number of cells analyzed. p values (two-tailed t-test) are shown above the corresponding comparisons.

bioRxiv preprint doi: https://doi.org/10.64898/2026.07.28.740790; this version posted July 29, 2026. The copyright holder for this preprint (which was not certified by peer review) is the author/funder, who has granted bioRxiv a license to display the preprint in perpetuity. It is made available under a CC-BY 4.0 International license.

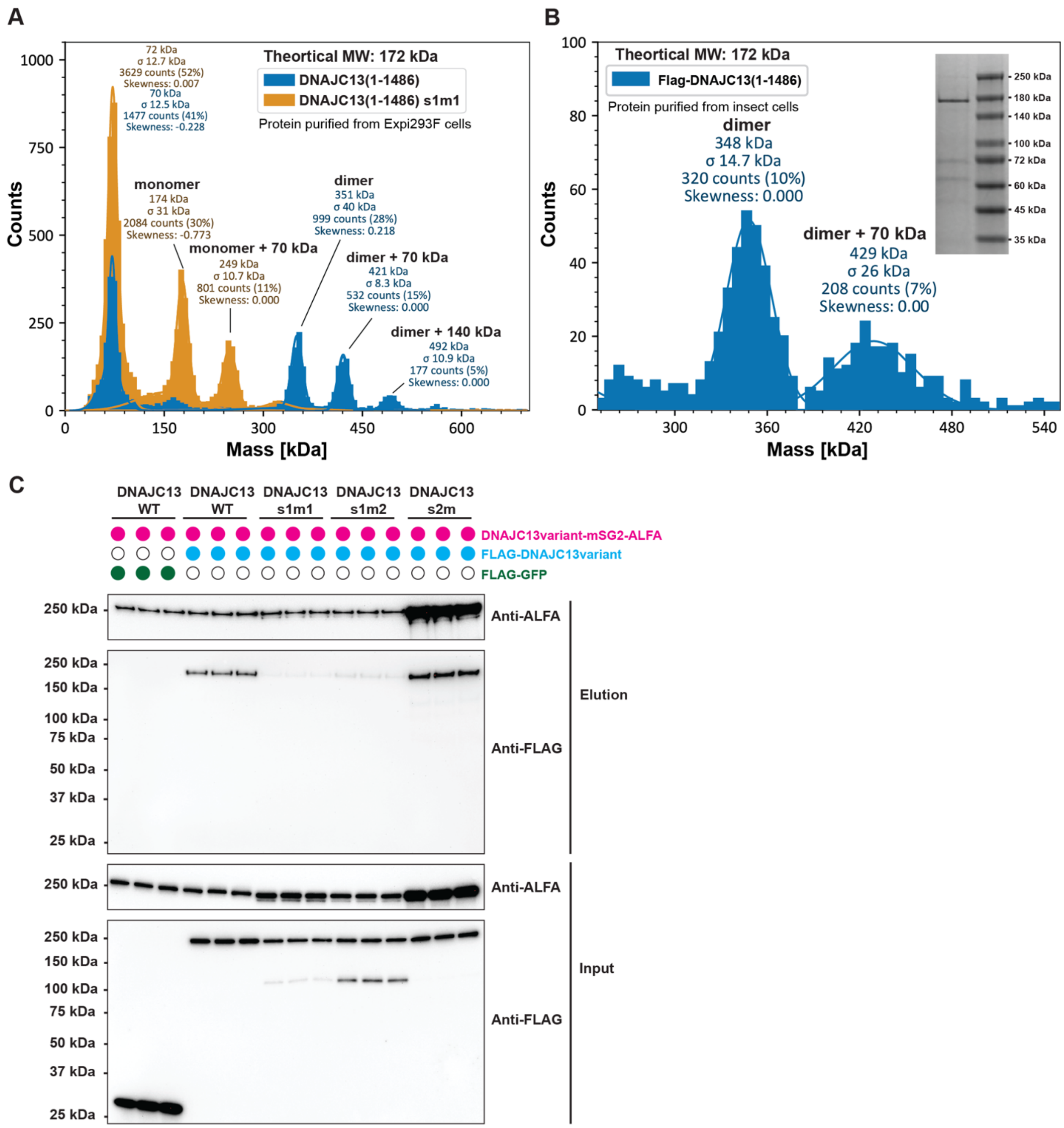

Figure S4. Mass photometry and co-immunoprecipitation demonstrate that DNAJC13 forms a stable homodimer, related to Figure 4.

(A) Mass photometry of Flag-DNAJC13(1-1486) (orange) and the site-1 interface mutant s1m1 (blue) purified from Expi293F cells. DNAJC13(1-1486) (theoretical monomer mass 172 kDa) is observed as dimer, and dimer plus one or two 70-kDa binding partners. In contrast, s1m1 produces peaks corresponding almost exclusively to monomer and monomer plus a 70-kDa complex, indicating that s1m1 disrupted dimerization.

(B) Mass photometry of Flag-DNAJC13(1-1486) purified from insect cells. A predominant peak at 348 kDa is consistent with a dimer, with a smaller population of dimer plus a 70-kDa binding partner. Inset, Coomassie-stained SDS-PAGE of the protein.

(C) Pull-down assay testing dimer formation of DNAJC13 variants in DNAJC13-KO HEK293T cells. ALFA-tagged DNAJC13-mSG2 variants were co-expressed with FLAG-tagged DNAJC13 variants or

bioRxiv preprint doi: https://doi.org/10.64898/2026.07.28.740790; this version posted July 29, 2026. The copyright holder for this preprint (which was not certified by peer review) is the author/funder, who has granted bioRxiv a license to display the preprint in perpetuity. It is made available under a CC-BY 4.0 International license.

FLAG-GFP (negative control, open circles) in HEK293T cells. Top panels: anti-ALFA and anti-FLAG immunoblot of proteins eluted from ALFA resin (Elution fraction). Bottom panels: corresponding input lysates. Equal volumes of input or eluate were loaded for all conditions.

bioRxiv preprint doi: https://doi.org/10.64898/2026.07.28.740790; this version posted July 29, 2026. The copyright holder for this preprint (which was not certified by peer review) is the author/funder, who has granted bioRxiv a license to display the preprint in perpetuity. It is made available under a CC-BY 4.0 International license.

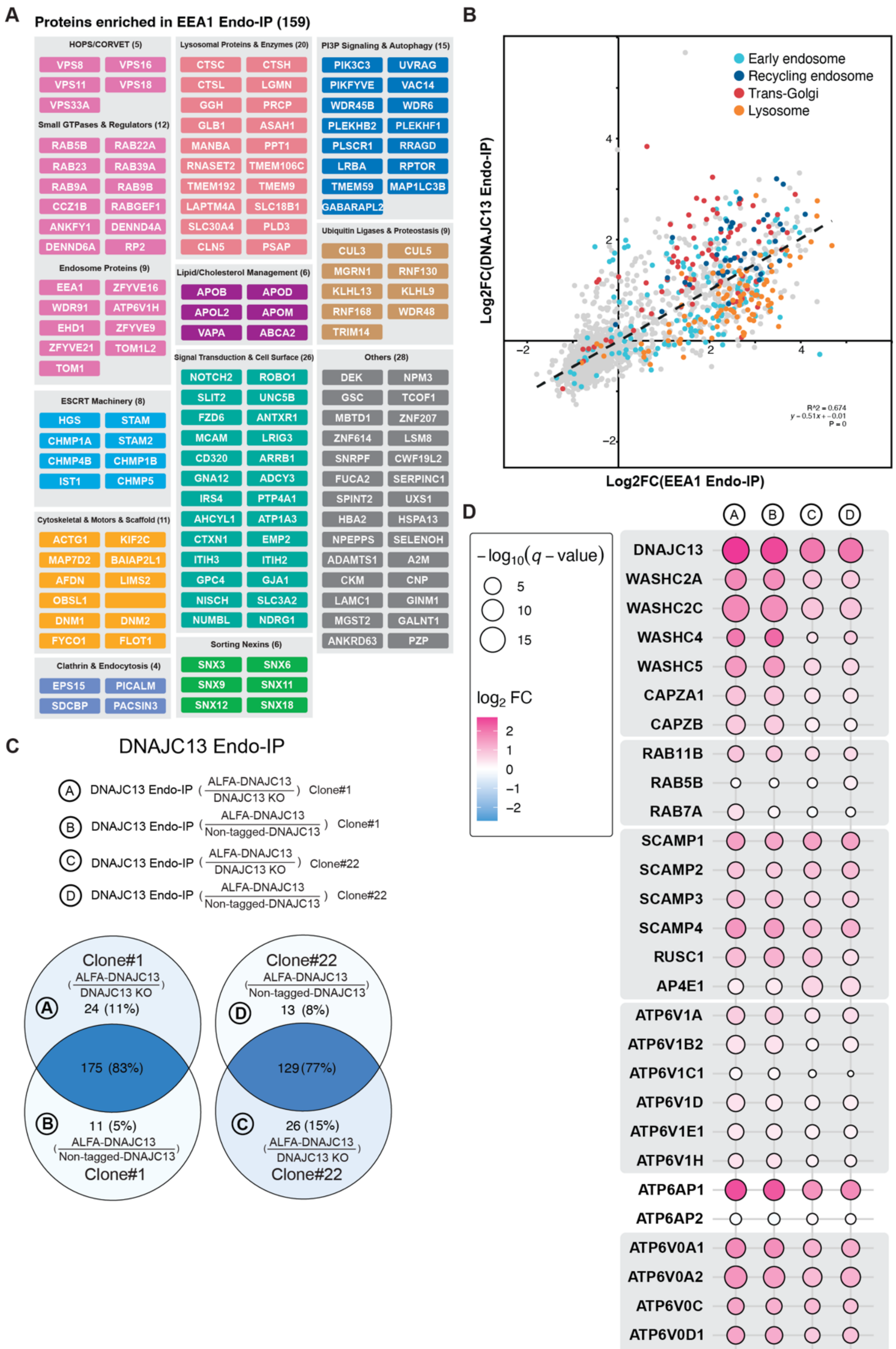

Figure S5. DNAJC13 Endo-IP preferentially enriches TGN and recycling-endosome components, related to Figure 5.

(A) Functional annotation of proteins selectively enriched in EEA1 Endo-IP (FC > 1.5, q < 0.01) as compared with DNAJC13 Endo-IP.

bioRxiv preprint doi: https://doi.org/10.64898/2026.07.28.740790; this version posted July 29, 2026. The copyright holder for this preprint (which was not certified by peer review) is the author/funder, who has granted bioRxiv a license to display the preprint in perpetuity. It is made available under a CC-BY 4.0 International license.

(B) Global comparison of protein enrichment between EEA1 Endo-IP and DNAJC13 Endo-IP. Each point represents a protein, plotted by its log_2 fold change in the EEA1 Endo-IP (x-axis) versus the DNAJC13 Endo-IP (y-axis). Proteins annotated as early endosome, trans-Golgi, recycling endosome, or lysosome (as employed previously[45]) are highlighted in cyan, red, navy, and orange, respectively; all other proteins are shown in gray. The dashed line shows the linear regression across all proteins, with the regression equation, R^2, and P value indicated.
(C, D) Reproducibility across DNAJC13 Endo-IPs using either Clone #1 or #22 cells expressing ALFA-tagged DNAJC13 with or without expression of untagged DNAJC13, as outlined in Panel C. Panel D shows a bubble heat map comparing the relative enrichment of selected proteins in the various DNAJC13 Endo-IP samples from Panel C. Circle color indicates log_2 fold change and circle size represents $-log_{10}$(q-value). Proteins include WASH complex subunits and partners, V-ATPase subunits, recycling-endosome markers (RAB11B), and TGN-endosomal trafficking factors (RUSC1/2, AP-4 subunits).

bioRxiv preprint doi: https://doi.org/10.64898/2026.07.28.740790; this version posted July 29, 2026. The copyright holder for this preprint (which was not certified by peer review) is the author/funder, who has granted bioRxiv a license to display the preprint in perpetuity. It is made available under a CC-BY 4.0 International license.

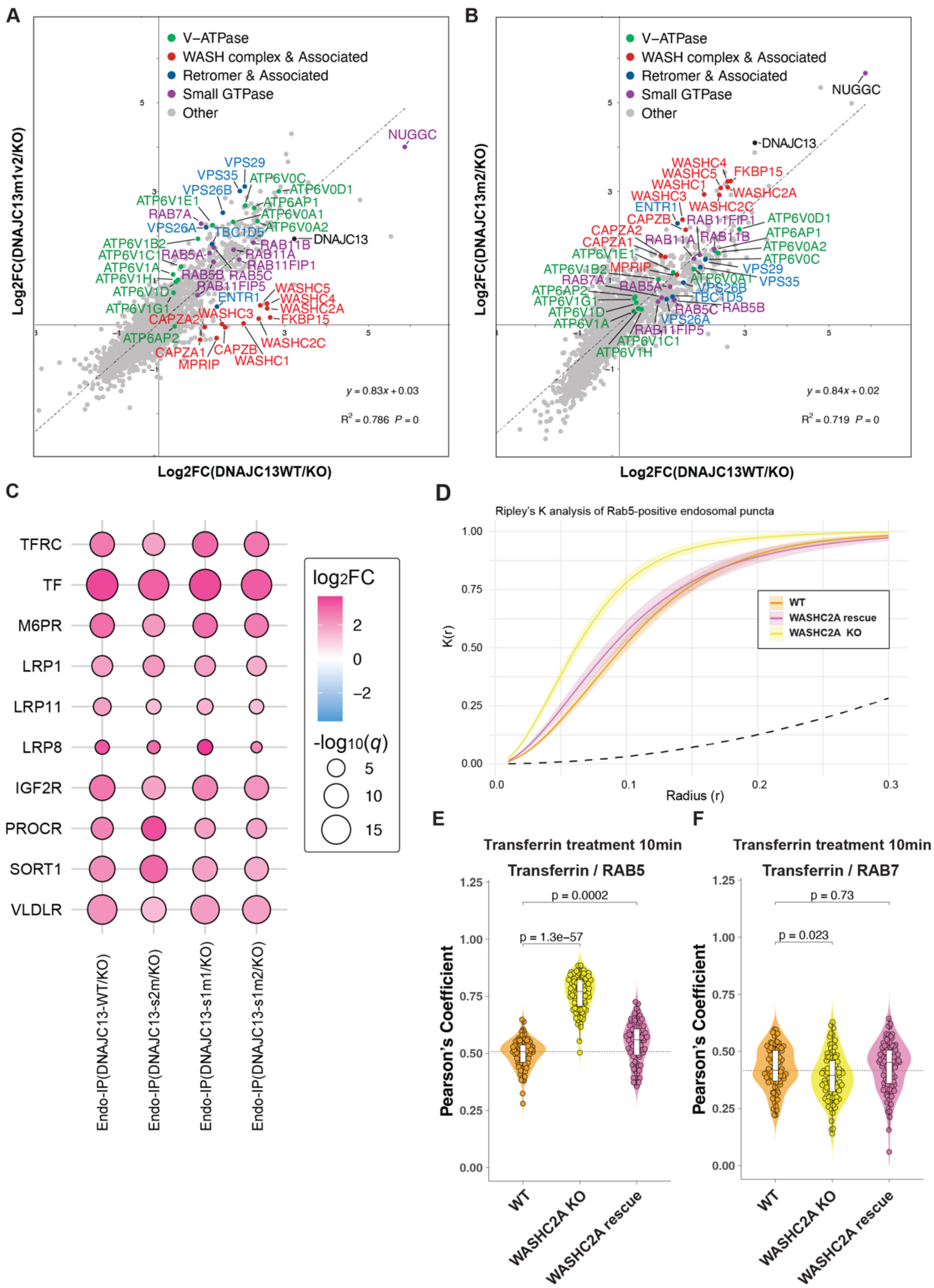

Figure S6. DNAJC13 dimerization is required for the WASH complex and V-ATPase assembly, related to Figure 6.

(A, B) Comparison of DNAJC13 Endo-IP proteomes obtained with DNAJC13-WT/KO versus the dimerization-defective mutants DNAJC13-s1m1/KO (A) or DNAJC13-s1m2/KO (B). Scatter plots show $\log_2$ fold changes (WT/KO, x-axis) versus $\log_2$ fold changes (s1m1/KO or s1m2/KO, y-axis) for all detected proteins. V-ATPase subunits (green), WASH complex and associated proteins (red), retromer and

bioRxiv preprint doi: https://doi.org/10.64898/2026.07.28.740790; this version posted July 29, 2026. The copyright holder for this preprint (which was not certified by peer review) is the author/funder, who has granted bioRxiv a license to display the preprint in perpetuity. It is made available under a CC-BY 4.0 International license.

associated proteins (blue), and small GTPases (purple) are highlighted. Linear-regression fits, equations, R^2, and P values are shown.
(C) Bubble heatmap summarizing enrichment of individual V-ATPase subunits in DNAJC13 Endo-IPs. Data are shown for two clonal lines (Clone#1 and Clone#22) expressing DNAJC13-WT, using non-tagged DNAJC13 or DNAJC13 KO cells as controls. Circle color indicates $\log_2$ fold change, and circle size reflects the $-\log_{10}$(q-value), illustrating the consistent enrichment of a greater number of V_0 subunits compared to V_1 subunits in DNAJC13-positive endosomes.
(D) Ripley's K(r) was computed from the centroid coordinates of Rab5 puncta within single-cell ROIs for the indicated groups. For each ROI, puncta coordinates were linearly normalized to a common global bounding box (0-1 in x and y), and K(r) was calculated across radii r = 0.01–0.3 in normalized units (area fixed to 1). Bold lines and shaded ribbons show the group mean ±95% confidence interval. The dashed line indicates the expectation under complete spatial randomness (CSR, $K(r) = \pi r^2$), such that values above the dashed line reflect spatial clustering of Rab5-positive endosomal puncta relative to CSR.
(E, F) Quantification of Transferrin colocalization with RAB5 (D) or RAB7 (E) in WT, WASHC2A-KO, and WASHC2A-rescued cells after 10-min Transferrin uptake. Violin plots show the distribution of Pearson's coefficients for Transferrin/Rab5 or Transferrin/Rab7 colocalization; median and interquartile ranges are indicated. n denotes the number of cells analyzed, and p values (two-tailed t-test) are shown above the corresponding comparisons.

bioRxiv preprint doi: https://doi.org/10.64898/2026.07.28.740790; this version posted July 29, 2026. The copyright holder for this preprint (which was not certified by peer review) is the author/funder, who has granted bioRxiv a license to display the preprint in perpetuity. It is made available under a CC-BY 4.0 International license.

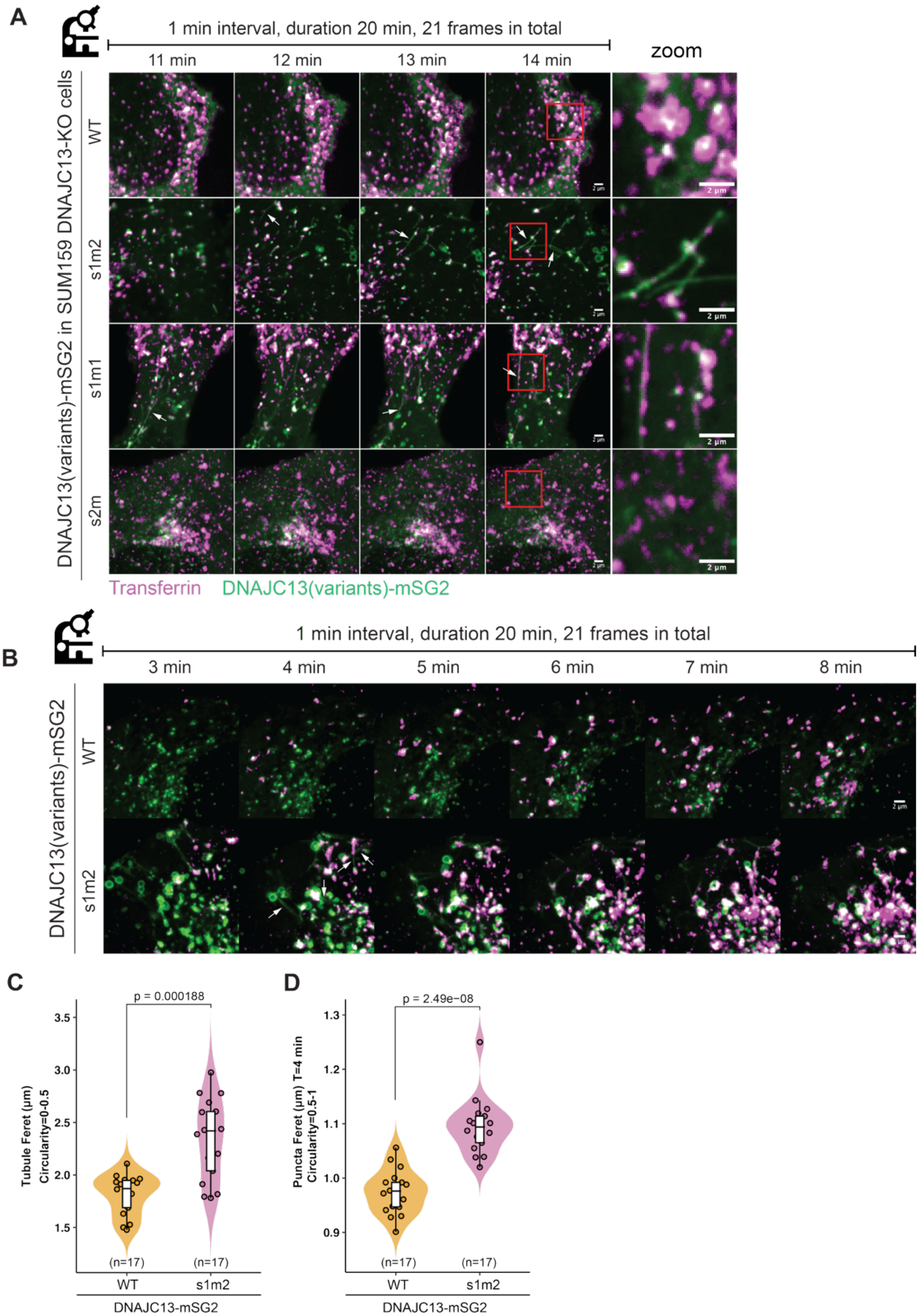

Figure S7. DNAJC13 dimerization controls endosomal tubulation

(A) Time-lapse imaging of DNAJC13$^{-/-}$ SUM159 cells expressing DNAJC13-mSG2 variants (green) following fluorescent Transferrin uptake (magenta). DNAJC13-WT, the dimeric mutant (s2m), and the monomeric mutants (s1m1 and s1m2) were imaged at 1-min intervals for 20 min; representative fields from

bioRxiv preprint doi: https://doi.org/10.64898/2026.07.28.740790; this version posted July 29, 2026. The copyright holder for this preprint (which was not certified by peer review) is the author/funder, who has granted bioRxiv a license to display the preprint in perpetuity. It is made available under a CC-BY 4.0 International license.

11-14 min are shown, with zoomed views of the regions outlined at 14 min on the right. Dimeric DNAJC13 (WT and s2m) predominantly decorates punctate endosomes, whereas monomeric variants (s1m1 and s1m2) form branched, highly dynamic DNAJC13- and Transferrin-positive tubules that emanate from endosomes and fail to undergo efficient fission. The red box in time 14 indicates the region shown in the rightmost panels. Scale bars, 2 µm. Additional views from the same experiment are shown in **Figure 7B**.
(B-D) Independent time-lapse experiment in DNAJC13$^{-/-}$ SUM159 cells expressing DNAJC13-mSG2 variants (green) following fluorescent Transferrin uptake (magenta), imaged at 1-min intervals for 20 min. (Panel B) Representative fields for DNAJC13-WT-mSG2 and DNAJC13-s1m2-mSG2 between 3 and 8 min. (C, D) Quantification of DNAJC13(variants)-mSG2-positive structures at t = 4 min, separated by object circularity into tubular (circularity 0-0.5) and punctate (circularity 0.5-1) populations. (Panel C) Violin plot show the distribution of tubule length (Feret diameters) for tubular objects (circularity 0-0.5) in WT and s1m2 cells. (Panel D) Violin plot show the distribution of puncta ferret diameter for punctate objects (circularity 0.5-1) in WT and s1m2 cells. N indicates the number of cells analyzed per group. Groups were compared using a two-sided Wilcoxon rank-sum test (Mann-Whitney U test) on Feret values and the p-values are shown above the plots.
