# Supplementary material for: Structural Mechanisms of DNAJC13 Dimeric Assembly and InsP6 binding in Recycling Endosome Regulation
Source: bioRxiv. 2026 Jul 29:2026.07.28.740790. Preprint. [Version 1] doi: 10.64898/2026.07.28.740790 (PMC13618431; doi:10.64898/2026.07.28.740790)

Raw images of western blots included in this study. Page 1/6

Raw data for Figure 2C and Data S2.4

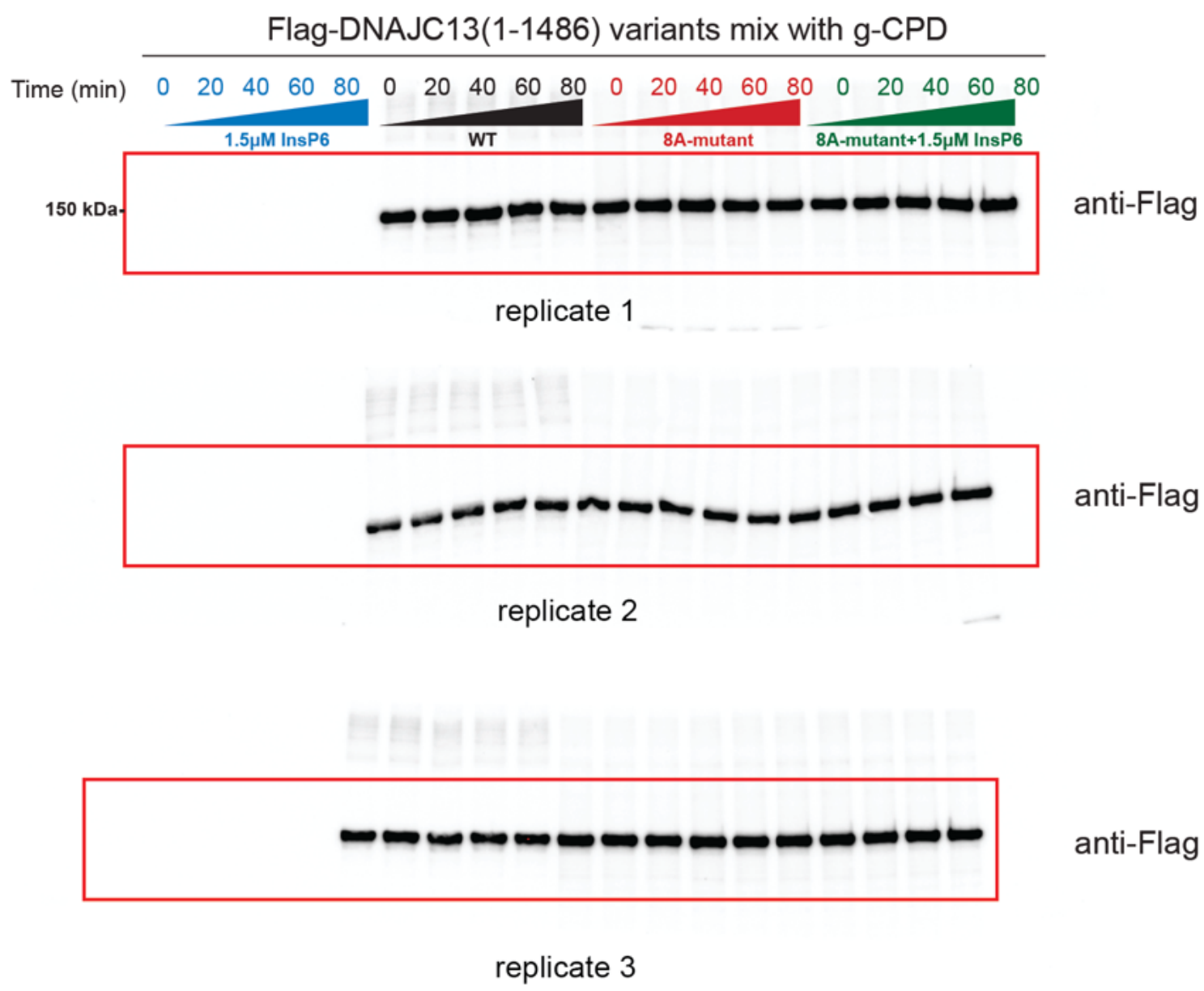

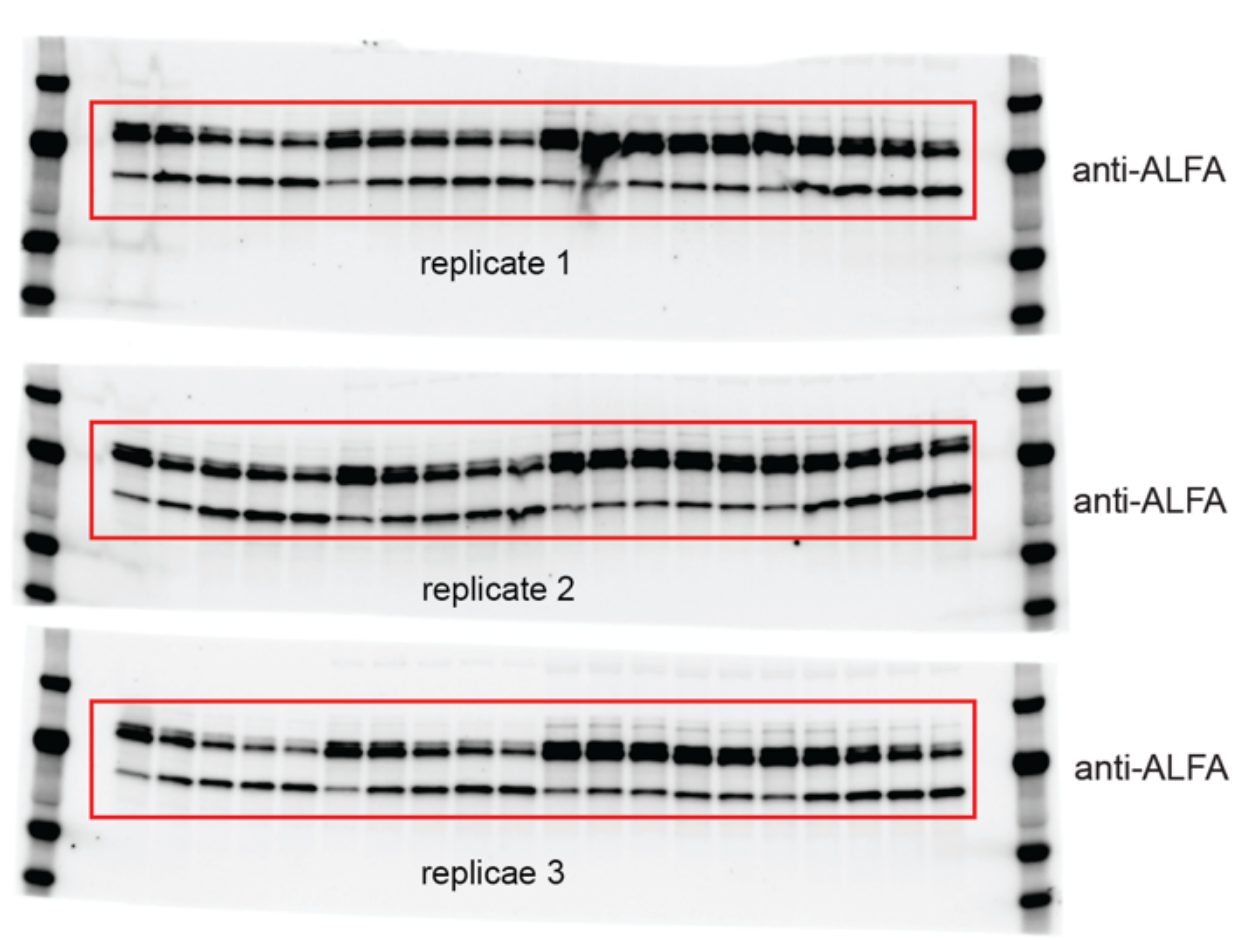

Raw images of western blots included in this study. Page 2/6

Raw data for Figure 7E

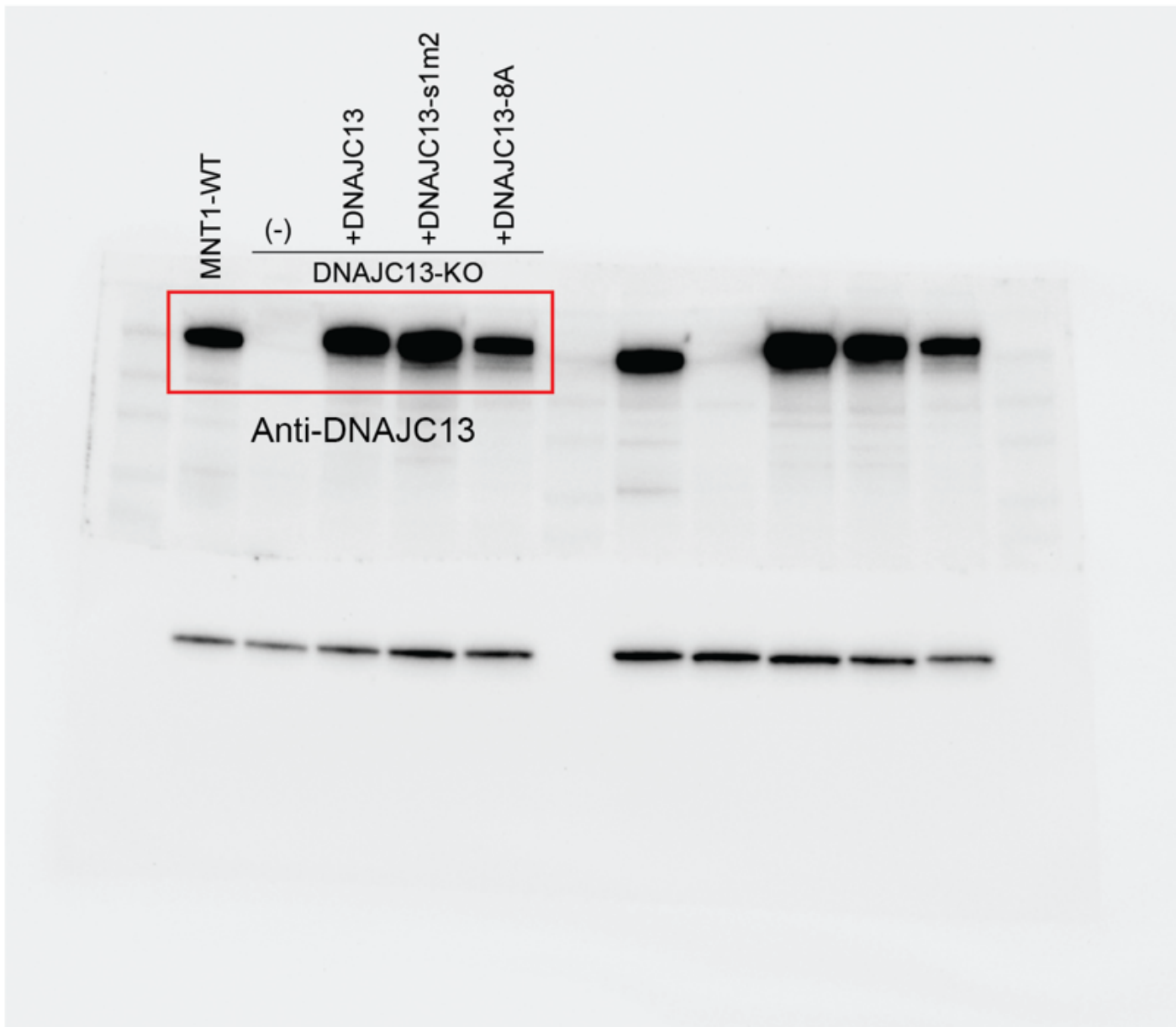

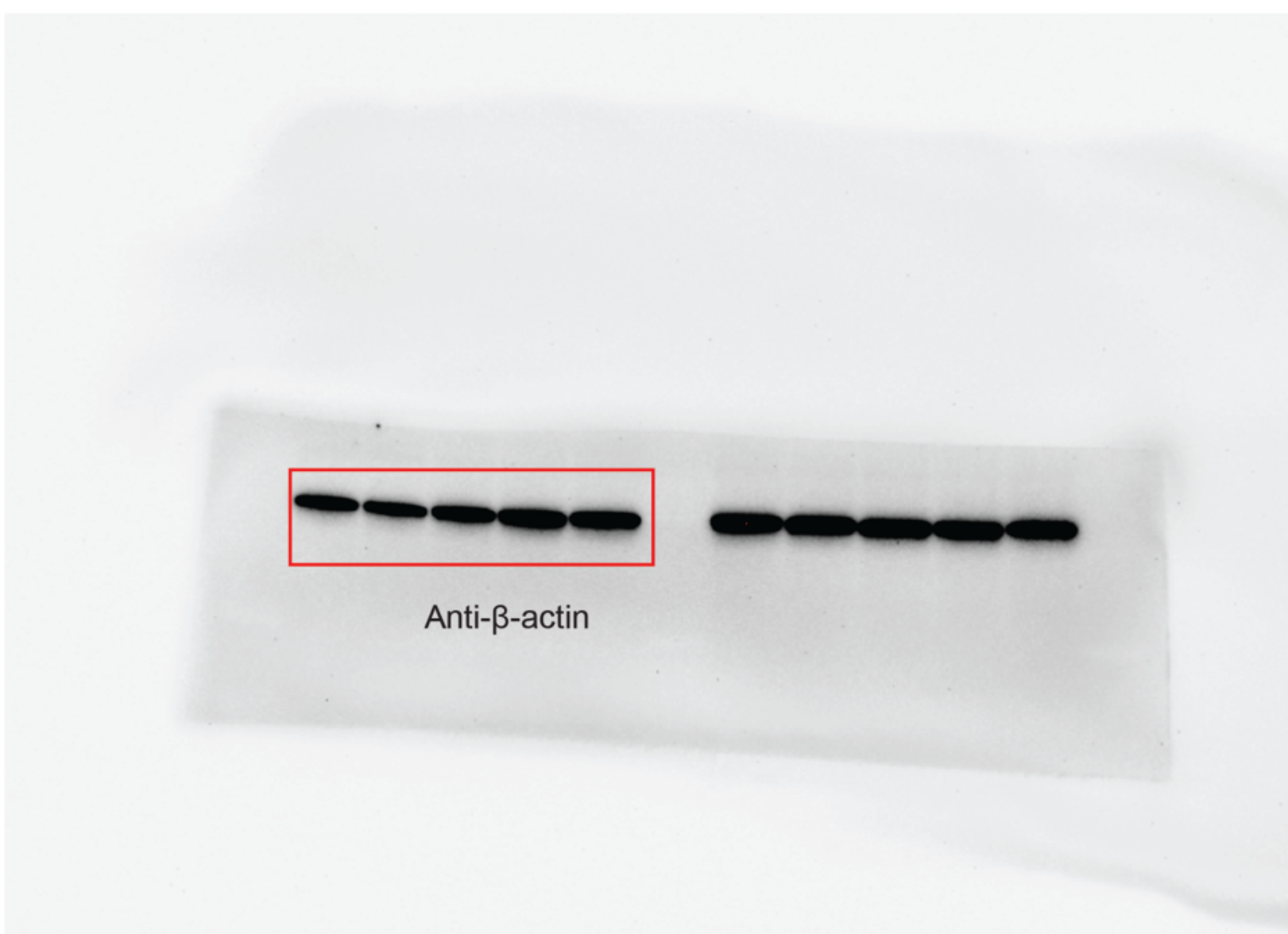

Raw images of western blots included in this study. Page 3/6

Raw data for Data S2.3C

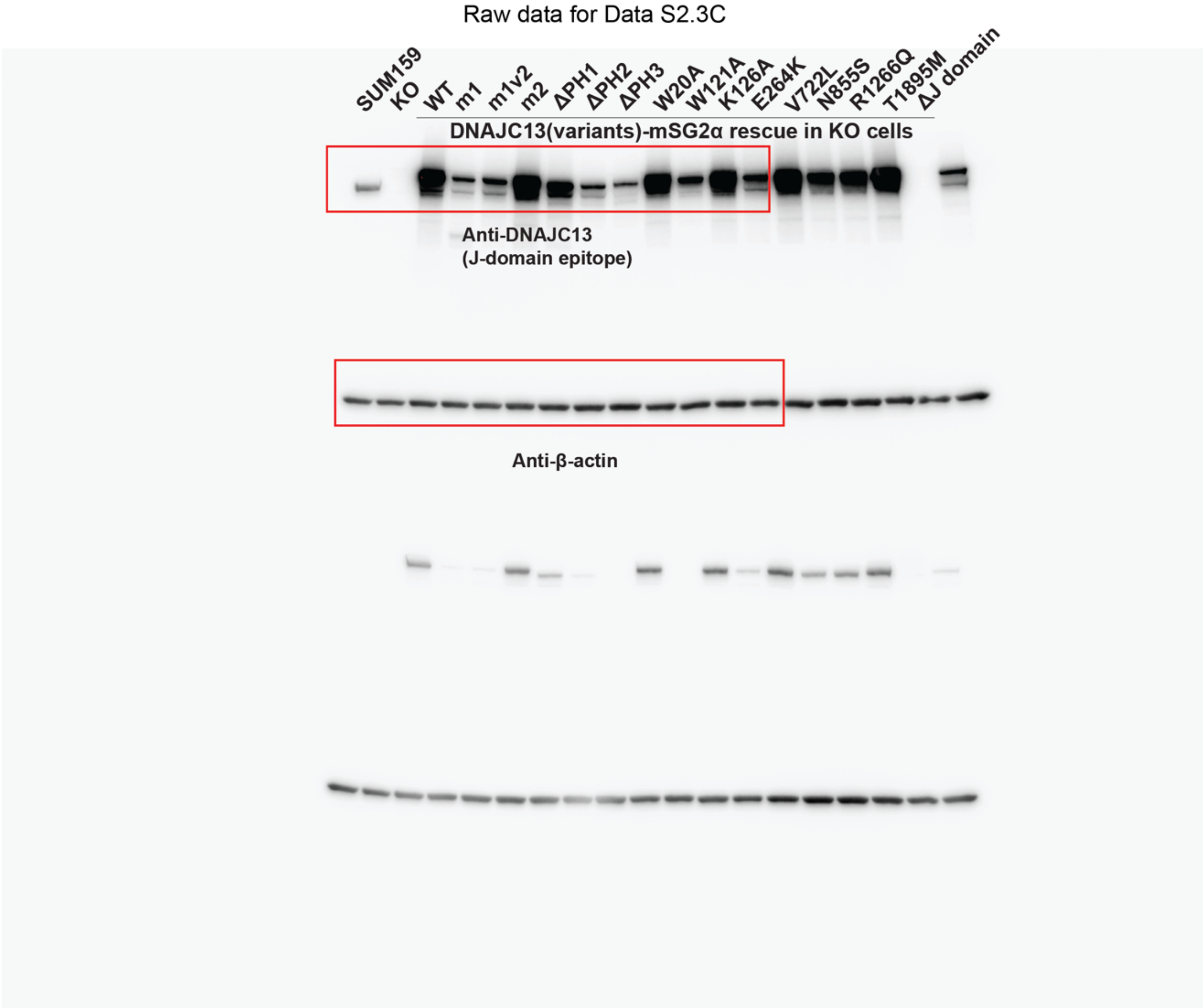

Raw images of western blots included in this study. Page 4/6

Raw data for Data S3A

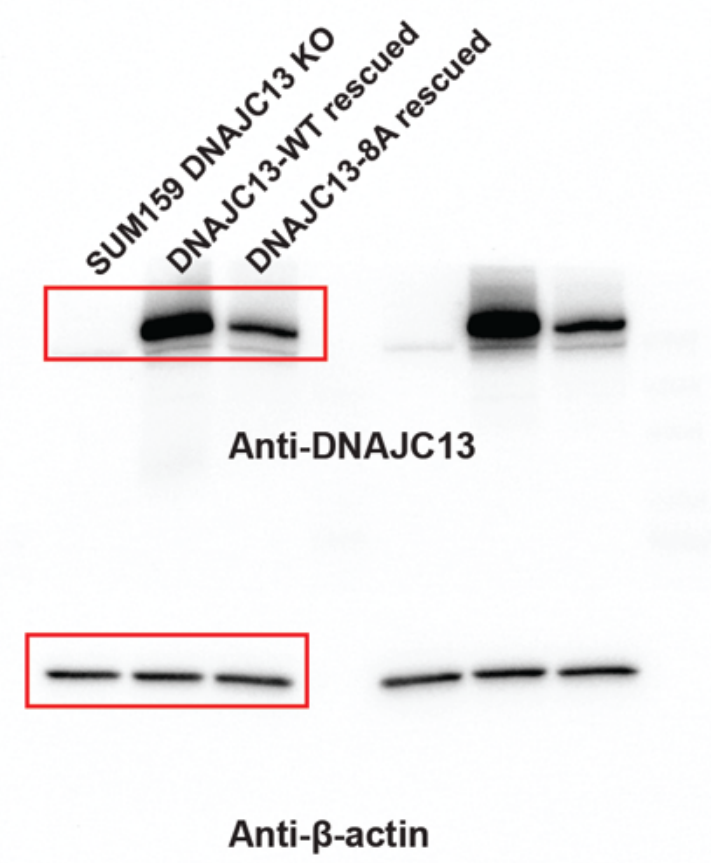

Raw data for Data S3B

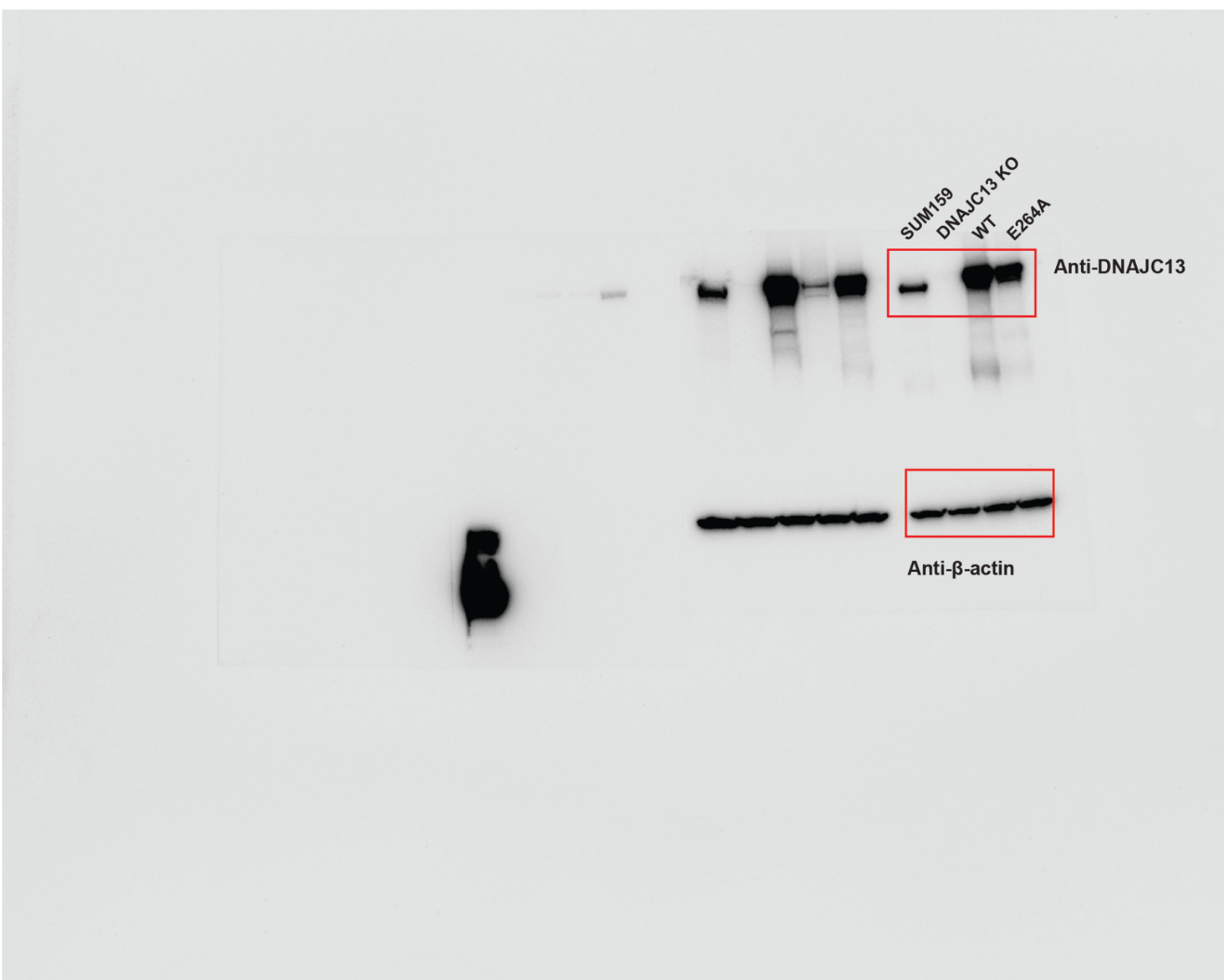

Raw images of western blots included in this study. Page 5/6

Raw data for Figure S3D

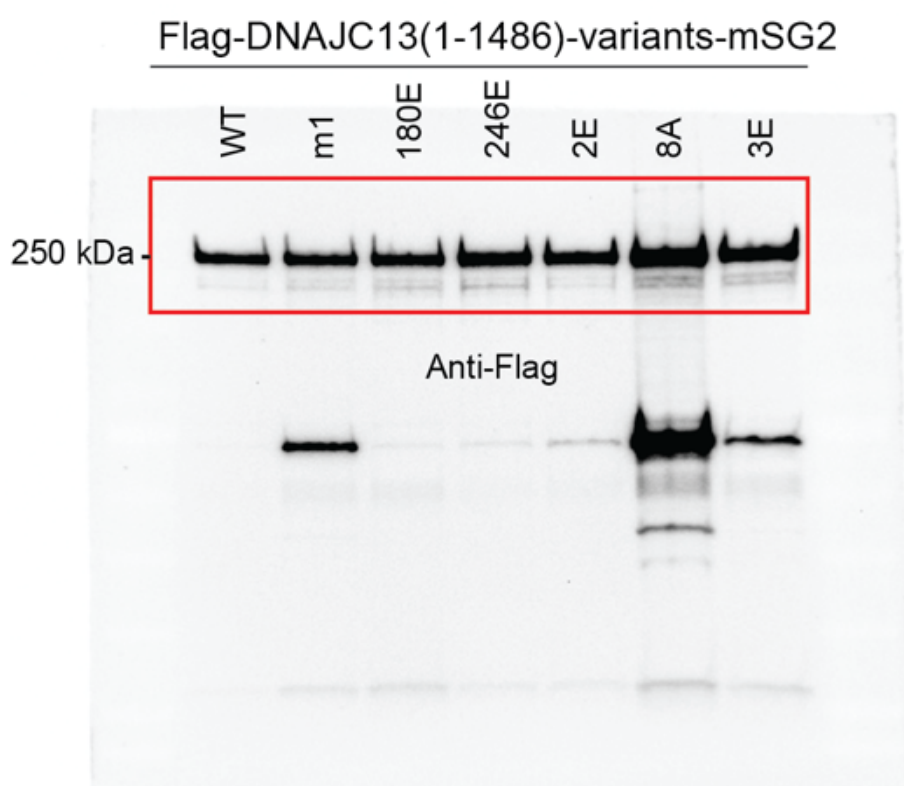

Raw data for Figure S4C

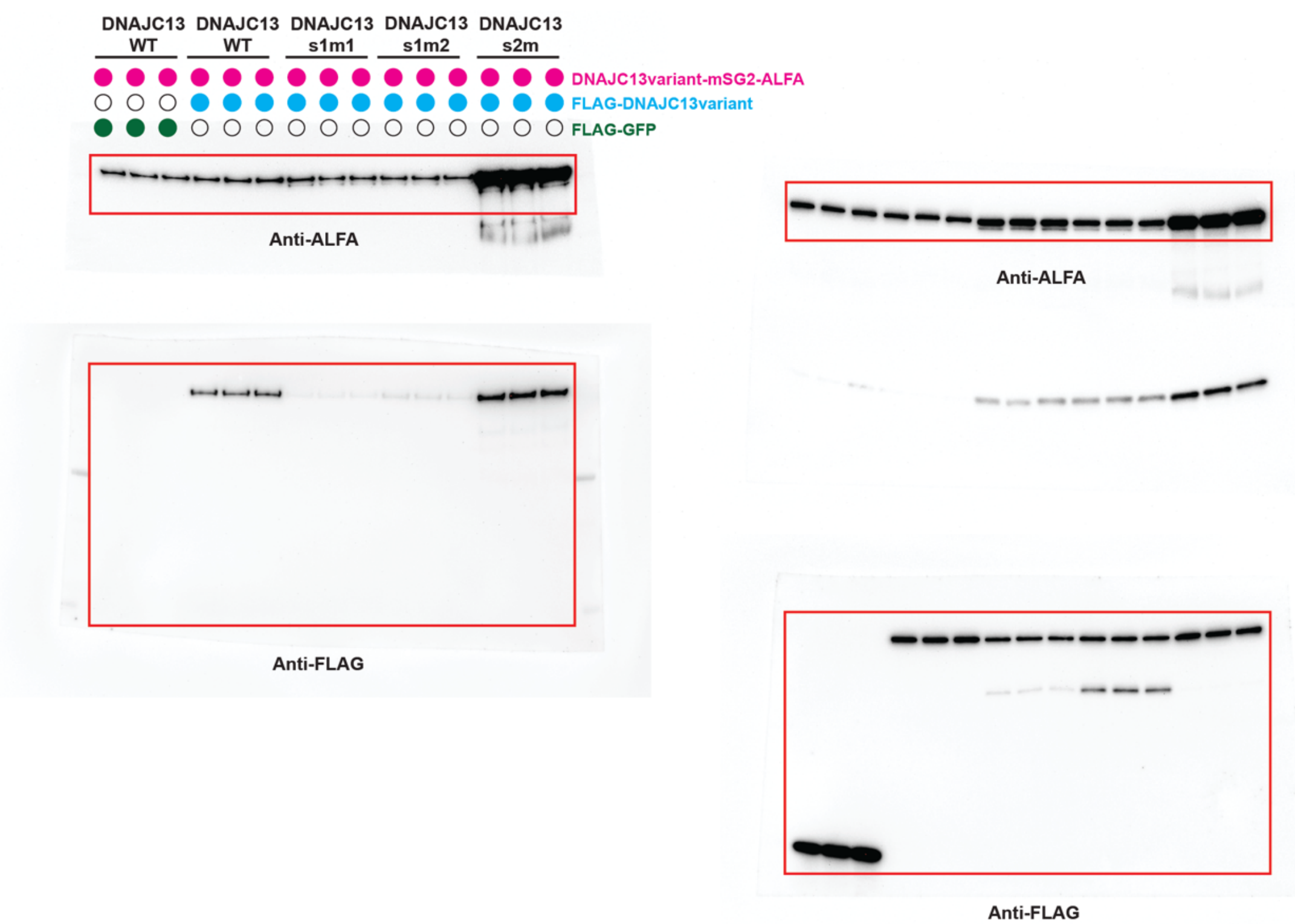

Raw images of western blots included in this study. Page 6/6

Raw data for Data S4

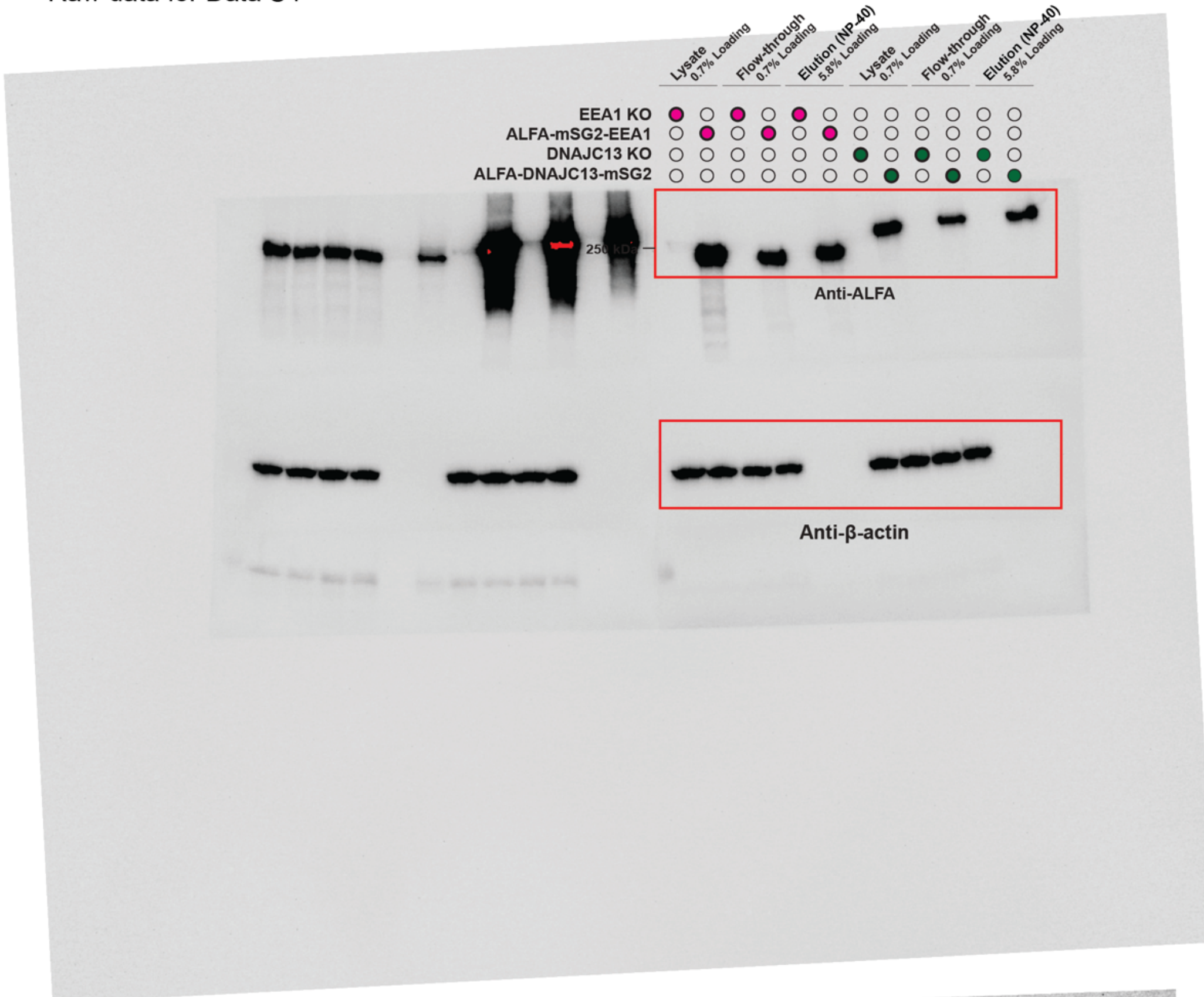

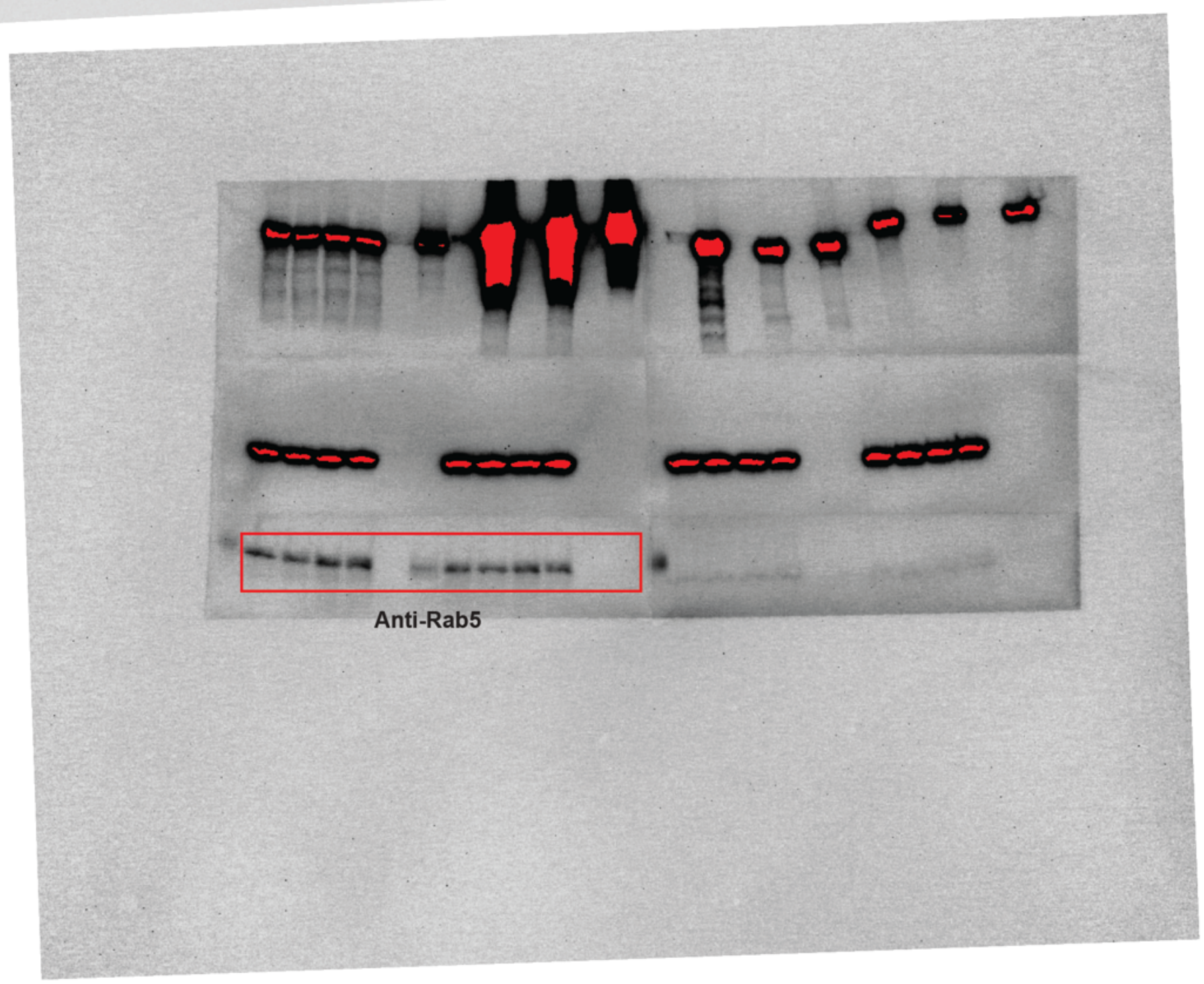

Supplement: Supplementary file 3 [file media-6.pdf]
